# Sensory Regulation of *Neuroligins* and *Neurexin I* in the Honeybee Brain

**DOI:** 10.1371/journal.pone.0009133

**Published:** 2010-02-09

**Authors:** Sunita Biswas, Judith Reinhard, John Oakeshott, Robyn Russell, Mandyam V. Srinivasan, Charles Claudianos

**Affiliations:** 1 Queensland Brain Institute, The University of Queensland, St. Lucia, Queensland, Australia; 2 Research School of Biological Sciences, Australian National University, Canberra, Australian Capital Territory, Australia; 3 Commonwealth Scientific and Industrial Research Organisation Entomology, Canberra, Australian Capital Territory, Australia; 4 Australian Research Council Centre of Excellence in Vision Science, Australian National University, Canberra, Australian Capital Territory, Australia; 5 School of Information Technology and Electrical Engineering, The University of Queensland, St. Lucia, Queensland, Australia; Centre de Recherches su la Cognition Animale - Centre National de la Recherche Scientifique and Université Paul Sabatier, France

## Abstract

**Background:**

Neurexins and neuroligins, which have recently been associated with neurological disorders such as autism in humans, are highly conserved adhesive proteins found on synaptic membranes of neurons. These binding partners produce a trans-synaptic bridge that facilitates maturation and specification of synapses. It is believed that there exists an optimal spatio-temporal code of neurexin and neuroligin interactions that guide synapse formation in the postnatal developing brain. Therefore, we investigated whether *neuroligins* and *neurexin* are differentially regulated by sensory input using a behavioural model system with an advanced capacity for sensory processing, learning and memory, the honeybee.

**Methodology/Principal Findings:**

Whole brain expression levels of *neuroligin 1–5* (*NLG1–5*) and *neurexin I* (*NrxI*) were estimated by qRT-PCR analysis in three different behavioural paradigms: sensory deprivation, associative scent learning, and lateralised sensory input. Sensory deprived bees had a lower level of *NLG1* expression, but a generally increased level of *NLG2–5* and *NrxI* expression compared to hive bees. Bees that had undergone associative scent training had significantly increased levels of *NrxI, NLG1* and *NLG3* expression compared to untrained control bees. Bees that had lateralised sensory input after antennal amputation showed a specific increase in *NLG1* expression compared to control bees, which only happened over time.

**Conclusions/Significance:**

Our results suggest that (1) there is a lack of synaptic pruning during sensory deprivation; (2) *NLG1* expression increases with sensory stimulation; (3) concomitant changes in gene expression suggests *NrxI* interacts with all neuroligins; (4) there is evidence for synaptic compensation after lateralised injury.

## Introduction

Sensory input arising from environmental stimuli, learning experiences, and social interactions manifests itself in-part through cell-to-cell contact of neurons via synapses. The neurexin/neuroligin adhesion system of synapses is highly conserved across species, even between vertebrates and invertebrates, although gene number and isoforms may vary [1]. Several compelling vertebrate studies highlight pre-synaptic neurexins and the post-synaptic neuroligins (trans-membrane cell adhesion binding partners) as critical to proper synapse development, specification and function [2], [3], [4], [5], [6], [7]. A number of studies have shown that the vertebrate neuroligin-neurexin complex appears to influence synapse specificity through excitatory versus inhibitory synapse development, and thus is predicted to influence the excitatory/inhibitory synapse ratio in the brain [8], [9], [10], [11], [12]. The combinatorial nature of neurexin/neuroligin interactions is believed to be key to neuronal plasticity mechanisms such as learning and memory, and also a likely mediator of mental disorders such as autism [6]. A mismatch of neurexin and neuroligin partners across synapses in the brain presumably leads to loss of synaptic plasticity and/or erroneous wiring, resulting in behavioural and cognitive deficiencies.

Recent *Drosophila NrxI* studies [13], [14], and our study in the honeybee [1] show that expression of *neuroligins* and *NrxI* is concentrated in the mushroom bodies. These anatomical structures are considered to be the higher order processing centres of the insect brain [15], [16], and suggested to be functionally analogous to the vertebrate hippocampus [17], [18]. Numerous *Drosophila* and honeybee studies have clearly illustrated the importance of the mushroom bodies in olfactory learning and integrating sensory information [15], [16], [19].

Despite possessing a small brain (one cubic millimetre comprising ∼950,000 neurons) honeybees display a broad and sophisticated behavioural repertoire in which sensory processing, learning and memory play a fundamental role in shaping and directing activities. In addition to displaying straightforward forms of learning, in which bees make specific associations between stimuli in their environment, bees can also master more complex tasks such as cross-modal associative recall, categorisation, contextual learning and rule abstraction, both in the visual and in the olfactory domain [19], [20]. Numerous assays have been formulated, in both controlled laboratory and field settings, which utilise this richness of experience-dependent behaviour in honeybees, to investigate the neural and molecular mechanisms underlying sensory processing, learning and memory [19].

The aim of our study was to assess whether the expression of *neuroligin(s)* and *NrxI* in the honeybee is associated with sensory input, sensory processing and learning. Three different paradigms were used. (1) The first paradigm was designed to observe whether *neuroligin* and *NrxI* expression is affected by sensory deprivation, and thus may play a role in synaptogenesis in response to environmental stimulation. This experiment was based on the long-standing and well documented observations that sensory deprivation profoundly affects the development of neuronal connectivity and has widespread consequences at cellular and behavioural levels. This association has been well established in all species possessing a central nervous system, from humans and rodents, through to invertebrates such as the bee and the nematode worm [21], [22], [23], [24]. (2) The second paradigm investigated a possible role of *neuroligins* and *neurexin* in associative learning, based on the observation that *Drosophila NrxI* null mutant larvae exhibit learning deficits [14]. In this paradigm, bees were subjected to associative scent training using the well-established proboscis extension reflex (PER) assay. The PER assay is a classical Pavlovian conditioning paradigm, which utilizes the fact that honeybees extend their proboscis (the insect tongue) in response to a sugar stimulus [25], [26], [27]. In the honeybee PER assay, an odour (conditioned stimulus, CS) is paired with a sugar reward (unconditioned stimulus, US), and the assay is used to assess how well associations are learned and memorized. (3) The third paradigm investigated the effect of lateralised sensory input on *neuroligin* and *neurexin* expression levels in the honeybee brain. Functional specialisation or lateralisation between the two hemispheres in the human brain is a recognised phenomenon [28], [29], [30], also observed in other vertebrate species [30], [31], [32], [33], [34], [35], [36], [37]. Recently, lateralisation has also been demonstrated in the honeybee in that they learn odours more effectively with their right antenna than with their left [38], [39] and colours more effectively with their right eye than with their left [40], indicating a dominance of sensory inputs from the right side in bees. To investigate a putative ‘synaptic’ role in lateralisation and brain development associated with sensory input, we examined honeybee *neuroligins* and *NrxI* expression levels in bees with the right or the left antennae amputated.

Taken together, our whole brain analyses show that *neuroligin* and *neurexin* expression is modulated substantially in a number of different contexts related to sensory processing.

## Results

### Sensory Deprivation

The first experiment examined whether the expression levels of honeybee *neuroligins* and *NrxI* are affected by sensory deprivation. A cohort of 100 bees was marked with blue paint on the thorax at emergence, caged with honey *ad libitum* as a food source, and kept in an incubator in the dark for the duration of the experiment. These sensory-deprived bees are termed *isolated bees* for the purposes of this study. Parallel to this, another 100 bees were marked with white paint at emergence and returned to the hive, representing the control bee cohort (*hive bees*). In the hive, bees are exposed to a plethora of sensory input such as olfactory and mechanical stimuli that are for the most part missing for the isolated bees. Hive bees also experience social interactions with other bees of all ages, including foragers who bring odorants, pollen and nectar samples from the outside. At later stages of development (2–3 weeks of age) hive bees begin to forage themselves and experience additional sensory input including visual and olfactory stimulation in the outside environment.

To assess potential developmental effects, sensory-deprived isolated bees and control hive bees were simultaneously collected as follows: 10 bees from each group at 24 hours, 7 days and 14 days of age. Brain tissue from these bees (10 isolated bees and 10 hive bees) was used for RNA analysis at each time point. Further time points could not be examined because nearly all sensory-deprived isolated bees had died by 21 days.

**Figure 1:**
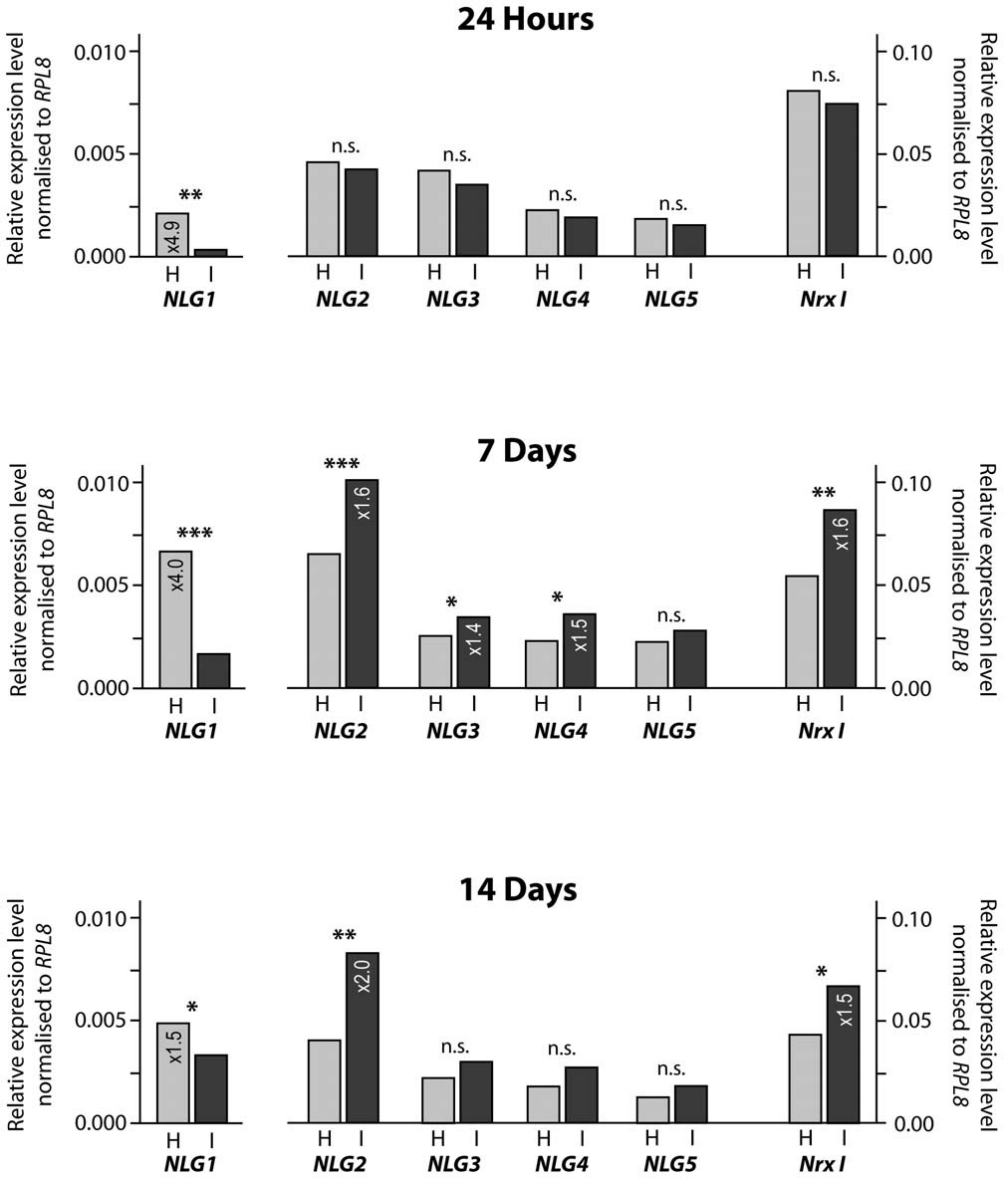
Effect of sensory deprivation during adult development on expression of *neuroligins* and *neurexin I* in honeybee brain. Expression of *neuroligins* and *neurexin I* in honeybee brain tissue, comparing bees that lived since emergence in a normal hive environment (H) with bees that lived since emergence in isolation in a dark incubator (I). Brain tissues were examined from bees aged 24 hours, 7 days and 14 days. *NLG1: neuroligin 1, NLG2: neuroligin 2, NLG3: neuroligin 3, NLG4: neuroligin 4, NLG5: neuroligin 5, NrxI: neurexin I*. Honeybee *neuroligin* and *neurexin I* expression was assessed by quantitative real time PCR amplification. The ribosomal gene *RPL8* was the housekeeping gene that was used as a reference level. Methodology for data analysis and the presentation of results was taken from Pfaffl [68] and Collins et al. [66]; whereby expression levels were normalised by subtraction against the threshold cycle of the *RPL8*. *RPL8* expression was equivalent in hive bees and isolated bees (standard deviation ±0.08–0.30 across all samples). Therefore, expression levels are shown relative to *RPL8*. *Neuroligin* and *neurexin* expression levels shown are means of three technical replicates each. Standards errors were negligible and less than ±1.29 for all experimental results. *** p<0.001, ** p<0.01, * p<0.05, n.s. no significant difference (*t*-test). Raw data from the qRT-PCR experiment are in Table S1.

Quantitative real time PCR amplification (qRT-PCR) identified changes to the levels of *NrxI* and *NLG* expression in sensory-deprived bees compared to the hive bees (Figure 1, Table S1). *NLG1*, which has generally a ten-fold lower relative expression level than the other highly expressed *neuroligins* and *neurexin* (up to 10% of ribosomal protein RPL8 expression [1]), showed the most significant difference in RNA levels between control hive bees and sensory-deprived isolated bees (Figure 1). The expression of *NLG1* was 4- to 5-fold greater in hive bees compared to isolated bees, at both 24 hours and 7 days of age. At 14 days of age, *NLG1* expression was still higher (1.5-fold) in hive bees than in isolated bees.

In contrast to *NLG1*, the expression of *NLG2–5* and *NrxI* showed a different profile over time. At 24 hours, expression levels of *NLG2–5* and *NrxI* were slightly lower in isolated bees compared to hive bees (Figure 1, Table S1). At 7 and 14 days the situation was reversed, with expression levels of *NLG2–5* and *NrxI* being increased in isolated bees (1.2-fold to 2.0-fold increase) compared to hive bees. These results show that sensory deprivation, as experienced by the isolated bees has a marked effect on *NrxI* and *neuroligin* expression levels in the brain. In addition to (or possibly because of) these environmentally elicited changes in expression of *neuroligins* and *NrxI*, sensory-deprived bees did not survive more than 14–21 days after emergence, in contrast to the hive bees, which lived for several weeks.

### Associative Scent Learning

**Figure 2:**
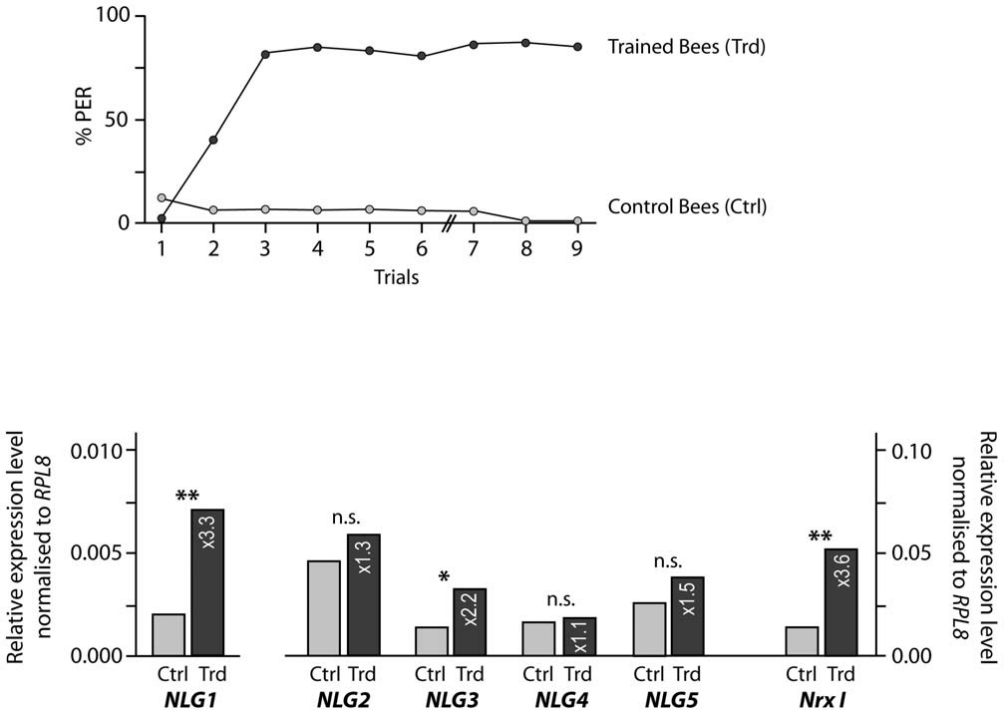
Effect of associative scent learning on expression of *neuroligins* and *neurexin I* in honeybee brain. Above: Acquisition of the Proboscis Extension Response (PER) by bees conditioned to associate lemon scent (CS) with a sugar reward (US) (Trained Bees), and bees that received the backward control (US presented before CS) (Control Bees). Nine trials were conducted over two days, using 23-day old bees. 20 bees per group were tested (for details see Materials and Methods). Below: Expression of *neuroligins* and *neurexin I* in honeybee brain tissue comparing trained bees (Trd) with control bees (Ctrl). *NLG1: neuroligin 1, NLG2: neuroligin 2, NLG3: neuroligin 3, NLG4: neuroligin 4, NLG5: neuroligin 5, NrxI: neurexin I*. Expression levels were assessed by quantitative real time PCR amplification; the detailed methodologies are as described in the legend to Figure 1. Expression levels are shown relative to *RPL8* (*RPL8* expression was equivalent in all experimental cohorts of bees: standard deviation ±0.03–0.30 across all samples). *Neuroligin* and *neurexin I* expression levels shown are means of three technical replicates each. Standard errors were negligible and less than ±1.22 for all experimental results. ** p<0.01, * p<0.05, n.s. no significant difference (*t*-test). Raw data from the qRT-PCR experiment are in Table S2.

The second experiment investigated whether the process of associative scent learning has an effect on the expression of honeybee *neuroligins* and *NrxI*. The proboscis extension reflex (PER) assay [27] was used to condition bees over two days to associate lemon scent (CS) with a sugar reward (US). 78% of the bees had learnt the association by trial 3 on the first day, and 80% of the bees showed a PER response to lemon scent on the second day (trials 7–9) (Figure 2), indicating they had formed a long-term memory of the association [19]. Another group of bees was subjected to a control treatment: they were restrained and kept in the same way as the trained bees and received the same amount of sensory exposure, but the sugar reward (US) was presented *before* the scent (CS) (backward control), thus preventing associative learning of US and CS. We used bees collected at 21 days post-emergence, as this is an age when bees typically forage and associative learning of scents is vital. At the end of the two-day procedure the bees were 23 days old. Brain tissue from ten bees was used from each of the two cohorts (trained and control groups) for RNA analysis.

The qRT-PCR analysis showed that the expression levels of honeybee *NrxI* and *neuroligins* were generally higher in bees that had undergone associative scent training compared to the control bees, which had the same sensory exposure, but did not learn the scent-reward association (Figure 2, Table S2). There was significant up-regulation of *NrxI* (3.6-fold higher in trained bees), *NLG1* (3.3-fold higher in trained bees) and *NLG3* (2.2-fold higher in trained bees), with expression levels in the other *neuroligins* showing only slight increases.

### Lateralised Sensory Input

The third experiment investigated the effect of lateralised sensory input on the expression of honeybee *neuroligins* and *NrxI*. This experiment was based on the recent discovery that, as in vertebrates, honeybees display lateralization of brain function [38], [39], [40]. Of particular interest to our study were the discoveries that bees learn scents better when trained through their right antenna than when trained through their left, and that the olfactory performance of bees trained through the right antenna alone is equivalent to bees trained with both antennae exposed [38].

To examine the roles that *neuroligins* and *NrxI* might play in the observed olfactory learning asymmetry, we used bees that had either the right or the left antenna amputated. Three groups of 50 bees were assessed: (1) control bees with both antennae intact; (2) bees that had only the right antenna (left antenna amputated); (3) bees that had only the left antenna (right antenna amputated). All three groups were captured at emergence, had the left or right antenna amputated within half an hour of emergence, and were returned to the hive. Thus, the only difference between cohorts was their ability to sense olfactory stimuli through the left, right, or both olfactory pathways. Five bees from each cohort were simultaneously collected at 24 hours, 7 days and 14 days post-emergence. At each developmental time point, the brains from the five bees of each of the three cohorts were dissected for RNA analysis. A fourth group of bees had both antennae amputated, but bees from this cohort did not survive 24 hours post amputation. Interestingly, the groups that had one antenna amputated, be it the left or the right antenna, also showed an increased mortality rate compared to the control bees, with only 40% of antenna-amputated bees surviving until 14 days.

**Figure 3:**
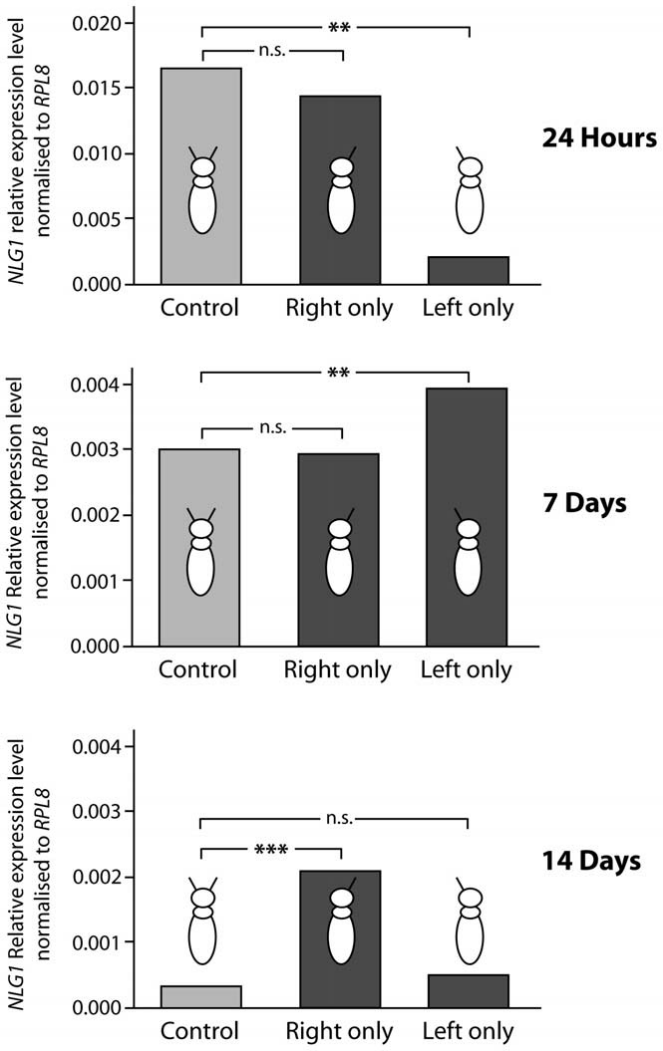
Effect of lateralised sensory input on expression of *neuroligin 1* in honeybee brain. Expression of *neuroligin 1* (*NLG1*) in honeybee brain tissue, comparing bees that had the left antenna amputated and the right antenna intact (Right only), bees that had the right antenna amputated and the left antenna intact (Left only), and bees that had both antennae intact (Control). Antennae were amputated immediately after emergence and the bees were returned to the hive. Brain tissue was examined from bees aged 24 hours, 7 days and 14 days, respectively (for details see Materials and Methods). Expression levels were assessed by quantitative real time PCR amplification; the detailed methodologies are as described in the legend to Figure 1. Expression levels are shown relative to *RPL8* (*RPL8* expression was equivalent in all experimental cohorts of bees: standard deviation ±0.06–0.10 across all samples). *Neuroligin 1* expression levels shown are means of three technical replicates. Standards errors were negligible and less than ±1.13 for all experimental results. *** p<0.001, ** p<0.01, n.s. no significant difference (*t*-test). Raw data from the qRT-PCR experiment are in Table S3.

The expression levels of *Neuroligins 2–5* and *NrxI* showed no substantial differences when comparing the control group and either of the antenna-amputated groups. Any observed differences were only in the 1.1-fold to 1.4-fold range, compared to the control cohort (Table S3). Lateralization of sensory input only had a significant effect on expression levels of *NLG1*. At 24 hours post-emergence, right-antenna-only bees showed a slight decrease in levels of *NLG1* expression compared to control bees, which had both antennae intact (Figure 3). This would support previous behavioural evidence that learning through the right antenna alone is equivalent to learning through both antennae [38]. In contrast, left-antenna-only bees had a significantly lower expression level of *NLG1* (7.5-fold less than the other two groups). This result is consistent with observations that learning performance is significantly worse when bees use only the left antenna [38].

At 7 days, the control bees and the right-antenna-only bees maintained an equivalent level of *NLG1* expression, while left-antenna-only bees now showed a 1.3-fold higher expression than the other two groups. At 14 days, the left-antenna-only bees and the control bees showed similar *NLG1* expression (slightly higher in left-antenna-only bees). However, the right-antenna-only bees now showed a significant (7.2-fold) increase in *NLG1* expression (Figure 3). These data suggest that there is a compensation mechanism associated with lateralized neural trauma possibly involving synapse development and neuronal rewiring. This apparent compensation occurs over time in the adult bee brain and is only evident when the dominant right side is unaffected.

### *Neuroligin 1* Alternative Splicing

Phylogenetic analyses indicate that *NLG1* is a putative common ancestor of the vertebrate and invertebrate *neuroligins* [1]. Its structural conservation over hundreds of millions of years arguably reflects a considerable degree of functional constraint. Interestingly, *NLG1* has a lower level of expression compared to *NLG2–5* in the honeybee brain [1]. This raises the question whether there are potential isoforms of NLG1 that might have subtly different functions in sensory processing. We therefore investigated alternative splicing of this gene. To identify putative splice variants, primers corresponding to the start and stop codon of full-length *NLG1* were designed for reverse transcription (RT) PCR amplification from brain cDNA. A sample size of over 50 RT-PCR amplicons arising from eight predominant RT-PCR products were cloned and sequenced. However, other than the full-length *NLG1* gene [1], no alternative splice variants of *NLG1* were identified. This is in contrast to the extensive alternative splicing that exists in other honeybee *neuroligins* and *NrxI* [1].

## Discussion

The aim of this study was to investigate the possible roles of *neuroligins* and *NrxI* in sensory processing. Our approach used three different paradigms: (1) sensory deprivation, (2) associative scent learning, and (3) lateralized sensory input. In all of the three situations, the qRT-PCR analyses show a significant effect of sensory input on *neuroligin* and *neurexin* expression levels in the honeybee brain. Below, we discuss the results of each experiment.

### Sensory Deprivation and Synapse Development

We see obvious changes in the expression of *NrxI* and the *neuroligins* in the brain between adult bees experiencing the normal sensory environment of the hive, compared to sensory deprived bees that where isolated at emergence and kept in a relatively impoverished sensory environment, with little social contact.

Although sensory processing logically depends on intrinsic properties of neurons specified via a developmental program, new sensory information needs to be processed in context to memory and motor control, and brain circuitry modified accordingly. Simple logic suggests that in the absence of sensory input the brain defaults to its developmental ground plan. A newly emerged adult bee relies on olfaction and tactile sensory input to recognise food, threat and relatedness in the dark environment of the hive. During this time the bee brain starts to wire up connections necessary for establishing vital memory important for social development [41]. The insect mushroom body structures are subject to experience- and age- related plasticity and are known to increase in volume throughout adult life [42], [43], [44]. This change in brain volume presumably reflects increased dendritic arborisation and synaptic development, driven by sensory experience. As pioneer synaptic proteins [7], neuroligins and neurexin are putatively useful markers for synaptogenesis and neural wiring.

Intuitively, we expected to see a down-regulation of *neuroligins* and *neurexin* in sensory-deprived bees. *NLG1* expression meets this expectation as it is consistently down-regulated in sensory deprived bees. This suggests that *NLG1* may specify synapses involved in regulating afferent neuronal circuits and neuromuscular motor control. Arguably, a lack of sensory stimulation reduces the requirement for excitatory cholinergic or glutamatergic synapses that are typically expressed in the brain and thorax and coincide with the tissue expression profile of *NLG1* [1], [45], [46], [47], [48].

In contrast, *NLG2–5* and *NrxI* were up-regulated in isolated bees at 7 and 14 days. One explanation for this result may be that bees kept in isolation at adult emergence forego synaptic or neuronal pruning via apoptotic process associated with normal postnatal/postembryonic brain development. That is, the brain of an isolated bee is kept in a pre-adapted or sensitive state to optimise afferent sensory input. The phenomenon of synaptic elimination has been reported in vertebrate models [49], [50], [51], [52], and has been associated with changes in the *Drosophila* eye [53] and antennal lobe [54], [55], but to our knowledge, our study is the first to suggest similar processes occur during adult brain development in the honeybee.

Sensory deprived bees did not live much beyond 14 days, in spite of abundant food supply. In contrast, all of the hive bees, which were exposed to a rich environment, lived to 21 days and longer. Although social context is arguably important for survival, our data suggests that sensory dependent synaptic development may be important for the brain to develop normally during early adulthood, and that exposure to a stimulating environment may be required for the normal longevity of a honeybee.

### Associative Learning and Gene Expression

Associative scent learning under controlled conditions in the laboratory has a substantial effect on expression levels of *NrxI* and *neuroligins*. A significant upregulation of *NLG1* (3.3-fold) and *NLG3* (2.2-fold) in trained bees compared to untrained bees supports a role for these molecules in synapse formation during learning and memory in 23-day-old forager bees. Considering the results from the sensory deprivation paradigm, *NLG1* in particular may have a role that is functionally distinct from those of the other honeybee *neuroligins*. An equivalent increase in the expression of *NrxI* after associative learning (3.6-fold) likely reflects the required interaction of these pre-and post-synaptic binding proteins in the synapse. The correlation of *NrxI* having a role in learning and memory processes in honeybees is consistent with results from *Drosophila* that show *NrxI* null mutant fly larvae exhibit clear deficits in associative learning [14].

We are mindful that 23-day-old forager bees are likely to have had natural foraging experience prior to our learning experiment. Foraging encompasses processing and learning of many new stimuli in the outside environment. Additional scent learning as in our experiment might have led to reinforcement or rewiring of existing foraging-related memories, as reflected by the increased *neuroligin* and *neurexin* levels in trained bees. Alternatively, the difference in expression levels between trained and control bees could be interpreted not as an increase in trained bees, but as a decreased expression in the control bees due to fading and extinction of their prior foraging memories, possibly linked with synaptic pruning.

The up-regulation of synaptic connectivity and brain development is a well-documented consequence of intensive learning and memory [56], [57]. Honeybee studies, specifically, have demonstrated an increase in neural development, particularly through mushroom body enlargement, as a consequence of sensory experience [42], [58], [59], [60], [61]. The concerted up-regulation of *NLG1, NLG3* and *NrxI* accompanying the formation of experience associative memories suggests these genes interact in similar molecular processes. Considering NLG3 and NrxI localisation [1], these processes likely involve synaptic changes in neurons associated with the olfactory (antennal lobes) and higher brain (mushroom bodies) centres in the bee.

### Lateralized Sensory Input and Regulatory Compensation

Lateralised sensory input through antennal amputation has only a marginal effect on expression levels of *NrxI* and *NLG2–5*, but a more pronounced effect on expression levels of *NLG1*. Whether *NLG1* is up- or down-regulated depends on the side of the sensory input as well as on the developmental age. Although these changes are subtle compared to the transcription levels of housekeeping genes like *RLP8* it does give us some insight how synapse development after injury in the honeybee may be associated with lateralised sensory dominance. A compelling aspect of our results is that they complement the findings of Letzkus et al. [38], which show that bees learn poorly with their left antenna and that the right-antennal pathway is necessary and arguably singularly sufficient for learning odours.

We did not formally examine the impact of infection or repair, other than to ascertain bees with amputated antennae showed no external features of infection or motor control impairment. Although the physiological impact of antennal amputation may be a contributing factor we are mindful that *NrxI* and *NLGs* are principally expressed in the postnatal/post-emergence brain [1] and are not obviously involved in early development or repair processes.

The results tempt us to consider the hypothesis, that there is a dominance of the right antenna for synaptic development associated with unilateral loss of sensory input. This is based on the following evidence: After loss of the right antennae (left-only bees) there is a significant perturbation in *NLG1* expression, with an immediate drop in *NLG1* RNA at 24 hours followed by an increase at 7 days falling to normal levels by 14 days, when compared to control bees. The brain seems to compensate for the loss of sensory input by increasing synaptic development and arguably new neural wiring associated with the unaffected side. This compensation mechanism works after loss of either antenna, but possibly represents an urgent response when the right, “dominant” antenna is missing compared to a slower compensation or adjustment in synaptic development at 14 days when the left antennae is removed (Figure 3).

We do not yet know where in the brain this compensatory expression occurs, that is whether *NLG1* expression increases in the ipsilateral, contralateral or equally in both hemispheres of the brain after antennal amputation. Either way, *NLG1* expression could be used as an effective marker for lateralization of neural wiring (and re-wiring) and of synaptic organization (and re-organization) during development, learning, and recovery from trauma. More immediately, it would be of interest to investigate the role of bee *NLG1* in the lateralization of vision [40]. Importantly, in addition to the results of the first two experiments, our lateralization data again highlight the participation of *NLG1* in processing sensory input in the honeybee and its distinct function as compared to *NLG2–5*.

### *Neuroligin 1*: Absence of Splice Variation

Our expression results coupled with behaviour suggest that *NLG1* expression is linked to sensory processing. This raised the question as to whether specific variants of *NLG1*, have subtly different functions. We therefore looked for splice variants of this gene. However, other than the full length *NLG1* gene [1], no apparent alternative splice variants of *NLG1* were identified. Given that alternative splicing is shown to be important to the specificity and function of vertebrate *neuroligins* and *neurexins* [62], [63], [64], and that extensive alternative splicing exists in other honeybee *neuroligins* and *NrxI* [1], this result suggests that *NLG1* has a unique role in sensory regulation. Barrow et al. [7] recently showed that axodendritic contact is closely followed by rapid accumulation of neuroligins that specifically interact and co-transport receptors such as NMDA (N-methyl-D-aspartic acid), thereby initiating formation and specification of synapses. If neuroligins indeed constitute a mechanism that specifies excitatory or inhibitory post-synaptic receptors, via selective interaction and co-transport, it would follow that a common single ancestral molecule such as *NLG1* likely specifies key synapses in the central nervous system. This ancestral function might have been tuned to sensory input in the brain and to motor control via neuromuscular signalling in other body parts. Aside from a putative role for NLG1 in the brain, characterising its role in the thorax and determining the possible existence of NGL1 isoform proteins in the thorax remains a priority.

### Conclusion

This study demonstrates that expression (up- and down-regulation) of *neuroligin* and *neurexin* genes is sensory-dependent during early adulthood. NrxI has a likely role as general interacting partner of the neuroligins, although the specific functions of putative invertebrate α- and β-neurexin isoforms need to be elucidated. NLG1 in particular has been highlighted as functionally distinct to other neuroligins, with its expression being tightly associated with sensory stimulation. Although remaining neuroligins are expressed at significant levels their expression is not obviously linked with sensory response possibly because these have modulating roles including specifying inhibitory synapses that act to filter or adapt sensory experience. Our findings support there is a functional conservation between vertebrate and invertebrate *neuroligins* and *neurexins*, where in humans they have been implicated in numerous cognitive disorders linked to sensory input. Importantly, this study shows how the honeybee can be used as a model species to examine sensory dependent plasticity in a whole brain.

## Materials and Methods

### Honeybees

Honeybees (*Apis mellifera*) were kept in an outdoor hive with abundant natural food sources. To obtain age-matched bees for the experiments, brood frames were collected from the hive and placed in an incubator at 32°C (80% humidity), until bees started to emerge from the cells. Newly emerged adult individuals were collected within 5 minutes of emergence, and marked with enamel paint on the thorax.

For the first paradigm (sensory deprivation) 100 bees of the same age, that is bees that had emerged more or less simultaneously, were marked with white paint and immediately returned to the hive (control bees). Another 100 bees of the same age were marked with blue paint, placed in a cage with honey *ad lib* as food source, and kept in the incubator in the dark at 32°C (80% humidity) for the duration of the experiment (isolated, i.e. sensory deprived bees). A group of 10 white bees from the hive and a group of 10 blue bees from the incubator were each collected at three distinct time points: 24 hours, 7 days, and 14 days after start of the experiment, i.e. post-emergence. They were immediately used for dissection of fresh tissue, as detailed below.

For the second paradigm (associative scent learning), another 100 bees were marked at emergence with paint and returned to the hive until collection. At 21 days, 40 bees were collected from the hive and subjected to a scent learning paradigm over two days using the Proboscis Extension Reflex Assay (PER), as described below. After completion of the scent-learning paradigm, that is when the bees were 23 days old, the bees were immediately used for dissection of fresh tissue.

For the third paradigm (lateralised sensory input), 200 bees were marked at emergence and had both, one, or no antennae removed as follows. 50 bees were marked with pink paint and had both antennae amputated with a sterile blade; 50 bees were marked with yellow paint and had the right antenna amputated; 50 bees were marked with green paint, and had the left antenna amputated; the last 50 bees were marked with blue paint and had both antenna intact, serving as control bees. All bees were returned to the hive. 5 bees from each group, as far as they had survived, were collected at 24 hours, 7 days and 14 days post-emergence and used immediately for dissection of fresh tissue.

### Proboscis Extension Reflex Assay (PER)

For the second paradigm (associative scent learning) bees were trained using the well-established PER Assay, during which bees learn to associate a scent with a sugar reward. Forty 21-day old bees were collected from the hive, and briefly immobilized in a refrigerator. They were then each mounted in a small plastic tube, secured with thin strips of GAFFA tape, so that the body, wings, and legs were immobile but the head was free to move. The restrained bees were fed to satiation with unscented 1 M sucrose solution and allowed to recover over-night in an incubator at 26°C (80% humidity). The next morning (day 22) they were separated in two groups of 20 bees each. The first group was subjected to PER conditioning pairing lemon scent (conditioned stimulus, CS) with 1 M sucrose solution (unconditioned stimulus, US). As scent we used Lemon Essence by Queens Fine Food Flavouring Essences. The second group of bees served as control. The control bees received the same sensory experience as the conditioned bees, but US and CS were presented backward (sugar presented before lemon scent), thus preventing associative learning of scent and sugar.

For the experimental procedure, the harnessed bees were put in front of an olfactory stimulus controller, which delivered a clean airstream through a syringe. The bees were placed into this airstream one at a time with their heads approximately 1.5 cm distant from the tip of the syringe. A second syringe containing a filter paper strip with 5 µl lemon scent was mounted next to the first syringe. To deliver a puff of scent, the airstream could be redirected through the second syringe via an electronic valve controlled remotely by the experimenter. A suction fan ensured quick removal of any lingering odour traces before the next bee was trained.

For conditioning, a bee from the first group was placed into the airstream, and lemon scent was presented to the bee for 6 s. Three sec after onset of the scent, the antennae were touched with a droplet of sugar solution exuding from the needle of a syringe, leading to extension of the proboscis. The bee was allowed to feed for 3 s, removed from the airstream, and the next bee was trained. Each bee was subjected to three such conditioning trials, separated by 6 min. The procedure for the control group differed only in the timing of sugar and scent presentation. The control bees first received a droplet of sugar, and after 120 sec lemon scent was delivered for 6 sec (backward control). The presentation of the sugar before the scent ensured that there was no associative learning and the long inter-stimulus interval prevented inhibitory conditioning [65]. Each bee in the control group was subjected to three such backward trials, separated by 6 min.

At the end of their three trials both the conditioned and the control bees were returned to the incubator for 3 hours. After 3 hours the entire procedure was repeated for both groups of bees. Afterwards they were fed with sugar solution to satiation and kept in the incubator over-night. The next morning (day 23) both groups of bees were subjected to another three trials each. Any bees that were sluggish, unresponsive or over-responsive during the trials were excluded from the experiment. The ten best learners from the conditioned group, that is bees that always extended their proboscis on presentation of lemon scent during trials 3–9, were selected, as well as ten bees from the control group that never responded to presentation of lemon scent. These 20 bees were used immediately for the next step, i.e. dissection of fresh tissue, and analysis of *neuroligin* and *NrxI* expression.

### Brain Dissection and RNA Extraction

Brain tissue was obtained by fresh tissue dissection. Ten bees from each cohort were used per time point in the first and second paradigms (i.e. sensory deprivation and associative scent learning), and five bees from each cohort at each time point (as far as available) in the third paradigm (lateralised sensory input). The live bees were placed in a freezer at −20°C for no longer than three minutes, and dissected as soon as feasible thereafter (up to five minutes). The head of each bee was removed using a sterile blade, followed by the frontal section of the head capsule, which was carefully removed to reveal the brain. The head was placed in an autoclaved petri dish in DEPC treated water. Glands were removed and the brain was gently prised out of the head under a microscope, using fine forceps that were sterilised and cleaned with RNase Zap (Ambion: #9780–9784). Brain tissue was then placed in autoclaved eppendorf tubes and kept on dry ice for immediate RNA extraction.

Total RNA was isolated using Trizol reagent (Invitrogen Life Technologies). 100 µl of Trizol was added to the 10–20 frozen brains and homogenised for 30 seconds. An additional 400 µl of Trizol was added, the tube inverted, and left for 5 minutes at room temperature. 100 µl of chloroform was added and mixed gently. The reaction was then spun for 10 minutes at 10,000 g. The upper phase was collected and mixed briefly with 400 µl of isopropanol. This was spun for a further 10 minutes at 12,000 g. The supernatant was removed carefully so as to not disturb the RNA pellet. The pellet was then washed with 500 µl of 75% ethanol. The adult brain RNA pellets from 5 or 10 brains were dissolved in 40 µl or 60 µl of distilled water, respectively. 2–4 µl of the RNA was then used for gel electrophoresis to assess the integrity of the extraction using a 1.5% Tris-Acetate-EDTA (TAE) gel made with diethyl pyrocarbonate (DEPC) treated water and run in DEPC-based TAE buffer. RNA samples were then quantified by spectrophotometry using a Nanodrop (Biolab: # ND1000; V3.2 software). RNA samples that were not used immediately were stored at −80°C in 0.1 volume of DEPC-based 3 M sodium acetate and 2.5 volumes of 100% ethanol (ready for RNA precipitation).

### Quantitative Real Time (qRT) PCR Amplification

For quantitative Real Time PCR (qRTPCR) amplification, the qRTPCR primer sequences for the housekeeping gene, *Ribosomal Protein L8* (*RPL8*), were taken from Collins et al. [66], who found *RPL8* to be the best correlate with total RNA concentration across varying honeybee developmental life stages and tissues. qRTPCR primers used to analyse the honeybee *NLG1–5* (NCBI: FJ580048, FJ580052, NM_001145736, NM_001145757, NM_001145739) and *NrxI* (NCBI: NM_001145740) expression were manually designed and then verified by PRIMER3 [67]. All primers were designed to work at similar annealing temperatures and to generate similar sized PCR amplicons. The primer sets were first checked with standard PCR amplification and gel electrophoresis (2.5% TAE, 25 bp ladder Promega: #19928601), and then used in a test qRTPCR experiment to assess primer specificity. All primer sets were highly gene specific and produced a single dissociation/melting (T_m_) curve. Table S1 from Biswas et al. [1] outlines the primer sets which were used, all at an annealing temperature of 55°C.

RNA was extracted as described above. 1 µg RNA was used in a 20 µL cDNA synthesis reaction, using the IScript cDNA synthesis kit (Bio-Rad #170–8891). cDNA samples were used at a dilution of 1∶20 for analysis of honeybee *NrxI* and *neuroligins*. The qRTPCR reactions were performed in triplicate and set up by an automated liquid handling instrument (Beckman Coulter: Biomek® 3000) into 96 well PCR plates (Bio-Rad: #2239441). Each reaction was 25 µls in total volume; composed of 10 µl 1∶20 cDNA sample and 15.0 µl master mix (12.5 µl ITaq SYBR Green Super-mix with ROX (Bio-Rad: #170–8850), 0.5 µl 10 µm forward primer, 0.5 µl 10 µm reverse forward primer, and 1.5 µl water). Each master mix was prepared immediately before the experiment and kept in darkness as much as possible. qRTPCR amplification was performed by the ABI Prism® 7000 Sequence Detection System (Applied Biosystems, 7000 SDS Instrument), software version 1∶2∶3. The relative quantification (ddCt) assay default settings were used, with the addition of an extra 15 second annealing step at 55°C. Relative quantification and standard deviation calculations were derived by the comparative method (outlined by Applied Biosystems). Methodology for final data analysis and the presentation of results was taken from Pfaffl [68] and Collins et al. [66], whereby expression levels were normalised by subtraction against the threshold cycle of *RPL8*.

### Statistics

For each experimental paradigm, differences in expression levels between treated and untreated bees as determined by (qRT) PCR were compared by *t*-tests: (1) hive bees vs isolated bees; (2) trained bees vs untrained bees; (3) control bees vs right-antenna-only bees and vs left-antenna-only bees. All comparisons were based on threshold cycle number itself, rather than transformed estimates of RNA quantity, because the former value best reflects experimental error.

### Reverse Transcription (RT) PCR Amplification

To identify putative splice variants of *NLG1*, primers corresponding to the start and stop codon of full-length *NLG1* were designed for reverse transcription (RT-PCR) amplification from brain cDNA. A sample size of over 50 RT-PCR amplicons, arising from eight predominant RT-PCR products, were cloned and sequenced. RT-PCR amplification was performed using the SuperScript III One-Step RT-PCR System with Platinum® *Taq* DNA Polymerase (Invitrogen: # 12574-018/026). Primers ‘*AmNLG1* Forward’ and ‘*AmNLG1* Reverse’ from Biswas et al. [1] were used with a 57°C-annealing step. The cDNA template used was from 12 day old adult honeybee brain, where *NLG1* is expressed [1]. The entire reaction was performed twice, and each resulting amplicon cloned and sequenced no less than five times. The RT-PCR amplicons were visualized using low melt TAE agarose gels and desired bands extracted for 10 µl in-gel ligation reactions performed overnight at 16°C, with the cloning vector pGEM®-T Easy (Promega: # A1380). 50 µl of distilled water was then added to each reaction and competent JM109 *E.coli* cells (Promega: #L2001) were chemically transformed with 10 µl of the diluted ligation reaction as per standard methods and grown overnight at 37°C on LB (Luria broth) agar media supplemented with ampicillin using standard (X-Gal/IPTG) blue/white selection. Single *E.coli* colonies were used to inoculate 10 ml of LB with 20 µl of 50 µg/µL ampicillin, and grown overnight at 37°C shaking. The plasmids were recovered using the QIAprep Spin Miniprep Kit (Qiagen: # 27104). Diagnostic restriction digests with *Eco*RI [69] were performed to check the fidelity of ligation. Plasmid DNA was then quantified by spectrophotometry with a Nanodrop (Biolab: # ND1000; V3.2 software) and sent for sequencing with M13 universal primers by the Australian Genome Research Facility (AGRF).

## Supporting Information

Table S1 Quantitative real time PCR data of *neuroligins* and *neurexin I* expression in isolated bees relative to hive (control) bees.
(0.50 MB PDF)

Table S2 Quantitative real time PCR data of *neuroligin* and *neurexin I* expression in PER trained bees (associative learning of scent with reward) relative to control bees (scent exposure without learning), using 23-day-old bees.
(0.31 MB PDF)

Table S3 Quantitative real time PCR data of *neuroligin* and *neurexin I* expression in bees with left or right antennal amputation.
(1.08 MB PDF)
