# Supplementary material for: Sensory Regulation of Neuroligins and Neurexin I in the Honeybee Brain
Source: PLoS One. 2010 Feb 9;5(2):e9133. doi: 10.1371/journal.pone.0009133 (PMC2817746; doi:10.1371/journal.pone.0009133)
Supplement: Table S1 — Quantitative real time PCR data of neuroligins and neurexin I expression in isolated bees relative to hive (control) bees. (0.50 MB PDF) [file pone.0009133.s001.pdf]

Table S1: Quantitative Real Time PCR Data of *Neuroligins* and *Neurexin I* Expression in Isolated Bees Relative to Hive (Control) Bees.

	TIME POINT	GENE	CONDITION	Relative (Fold) Expression to RPL8	Relative Expression as a ratio of RPL8		FOLD DIFFERENCE IN EXPRESSION BETWEEN HIVE AND ISOLATED BEES
		RPL8	**baseline**	***1**	**1.0000**		**1.000**
	24 HOURS	***NrxI***	HIVE	-12.452	0.0803	∨	1.103
	24 HOURS		ISOLATED	-13.737	0.0728		
	7 DAY		HIVE	-18.55	0.0539	∧	1.606
	7 DAY		ISOLATED	-11.55	0.0866		
	14 DAY		HIVE	-23.264	0.0430	∧	1.548
	14 DAY		ISOLATED	-15.032	0.0665		
	24 HOURS	***NLG1***	HIVE	-484.381	0.0021	∨	4.868
	24 HOURS		ISOLATED	-2357.976	0.0004		
	7 DAY		HIVE	-148.40	0.0067	∨	4.005
	7 DAY		ISOLATED	-594.28	0.0017		
	14 DAY		HIVE	-204.364	0.0049	∨	1.469
	14 DAY		ISOLATED	-300.246	0.0033		
	24 HOURS	***NLG2***	HIVE	-21.015	0.0476	∨	1.134
	24 HOURS		ISOLATED	-23.834	0.0420		
	7 DAY		HIVE	-15.49	0.0646	∧	1.606
	7 DAY		ISOLATED	-9.65	0.1037		
	14 DAY		HIVE	-12.295	0.0404	∧	2.012
	14 DAY		ISOLATED	-24.761	0.0813		
	24 HOURS	***NLG3***	HIVE	-22.523	0.0444	∨	1.344
	24 HOURS		ISOLATED	-30.273	0.0330		
	7 DAY		HIVE	-38.41	0.0260	∧	1.357
	7 DAY		ISOLATED	-28.31	0.0353		
	14 DAY		HIVE	-46.046	0.0217	∧	1.366
	14 DAY		ISOLATED	-33.708	0.0297		
	24 HOURS	***NLG4***	HIVE	-42.518	0.0235	∨	1.173
	24 HOURS		ISOLATED	-49.866	0.0201		
	7 DAY		HIVE	-41.31	0.0242	∧	1.504
	7 DAY		ISOLATED	-27.47	0.0364		
	14 DAY		HIVE	-51.804	0.0193	∧	1.454
	14 DAY		ISOLATED	-35.629	0.0281		
	24 HOURS	***NLG5***	HIVE	-48.784	0.0205	∨	1.228
	24 HOURS		ISOLATED	-59.921	0.0167		
	7 DAY		HIVE	-44.12	0.0227	∧	1.256
	7 DAY		ISOLATED	-35.14	0.0285		
	14 DAY		HIVE	-67.415	0.0148	∧	1.384
	14 DAY		ISOLATED	-48.728	0.0205		

*1 chosen as an arbitrary value of *RPL8* expression (housekeeping gene which all experimental genes were normalised against). Time point denotes time elapsed post emergence. *Neurexin I: NrxI. Neuroligin: NLG.* Symbols indicate if expression in hive bees is higher (∨) or lower (∧) than in the corresponding isolated bees.
