# Supplementary material for: Sensory Regulation of Neuroligins and Neurexin I in the Honeybee Brain
Source: PLoS One. 2010 Feb 9;5(2):e9133. doi: 10.1371/journal.pone.0009133 (PMC2817746; doi:10.1371/journal.pone.0009133)
Supplement: Table S2 — Quantitative real time PCR data of neuroligin and neurexin I expression in PER trained bees (associative learning of scent with reward) relative to control bees (scent exposure without learning), using 23-day-old bees. (0.31 MB PDF) [file pone.0009133.s002.pdf]

Table S2: Quantitative Real Time PCR Data of *Neuroligin* and *Neurexin I* Expression in PER Trained Bees (Associative Learning of Scent with Reward) Relative to Control Bees (Scent Exposure without Learning), Using 23-Day Old Bees.

GENE	CONDITION	Relative (Fold) Expression to *RPL8*	Relative Expression as a ratio of *RPL8*	FOLD DIFFERENCE IN EXPRESSION BETWEEN PER TRAINED AND CONTROL
**RPL8**		***1**	**1.0000**	
NrxI	23day PER Control	-68.77	0.0145	
	23day PER Trained	-19.17	0.0522	3.59
NLG1	23day PER Control	-472.48	0.0021	
	23day PER Trained	-143.55	0.0070	3.29
NLG2	23day PER Control	-21.46	0.0466	
	23day PER Trained	-17.07	0.0586	1.26
NLG3	23day PER Control	-68.754	0.0145	
	23day PER Trained	-31.24	0.0320	2.20
NLG4	23day PER Control	-57.81	0.0173	
	23day PER Trained	-50.94	0.0196	1.13
NLG5	23day PER Control	-37.66	0.0266	
	23day PER Trained	-25.55	0.0391	1.47

*1 was chosen as an arbitrary value of *RPL8* expression (housekeeping gene which all experimental genes were normalised against). *Neurexin I: NrxI. Neuroligin: NLG.*
