# Supplementary material for: Sensory Regulation of Neuroligins and Neurexin I in the Honeybee Brain
Source: PLoS One. 2010 Feb 9;5(2):e9133. doi: 10.1371/journal.pone.0009133 (PMC2817746; doi:10.1371/journal.pone.0009133)
Supplement: Table S3 — Quantitative real time PCR data of neuroligin and neurexin I expression in bees with left or right antennal amputation. (1.08 MB PDF) [file pone.0009133.s003.pdf]

Table S3: Quantitative Real Time PCR Data of *Neuroligin* and *Neurexin I* Expression in Bees with Left or Right Antennal Amputation

GENE	AGE	CONDITION	Relative (Fold) Expression to RPL8	Relative Expression as a ratio of *RPL8*	FOLD DIFFERENCE IN EXPRESSION BETWEEN AMPUTEES AND CONTROLS
**RPL8**			***1**	**1.0000**	
NrxI	24 hours	Control (Both Antennae Intact)	-15.02	0.0666	
	24 hours	Left Antenna Only (Right-Amputee)	-11.7	0.0855	1.28
	24 hours	Right Antenna Only (Left-Amputee)	-13.42	0.0745	1.12
NLG1	24 hours	Control (Both Antennae Intact)	-59.99	0.0167	
	24 hours	Left Antenna Only (Right-Amputee)	-452.47	0.0022	**7.54**
	24 hours	Right Antenna Only (Left-Amputee)	-70.85	0.0141	1.18
NLG2	24 hours	Control (Both Antennae Intact)	-15.85	0.0631	
	24 hours	Left Antenna Only (Right-Amputee)	-12.18	0.0821	1.30
	24 hours	Right Antenna Only (Left-Amputee)	-12.66	0.0790	1.25
NLG3	24 hours	Control (Both Antennae Intact)	-26.57	0.0376	
	24 hours	Left Antenna Only (Right-Amputee)	-27.7	0.0361	1.04
	24 hours	Right Antenna Only (Left-Amputee)	-23.95	0.0418	1.11
NLG4	24 hours	Control (Both Antennae Intact)	-29.58	0.0338	
	24 hours	Left Antenna Only (Right-Amputee)	-29.48	0.0339	1.00
	24 hours	Right Antenna Only (Left-Amputee)	-31.27	0.0320	1.06
NLG5	24 hours	Control (Both Antennae Intact)	-43.76	0.0229	
	24 hours	Left Antenna Only (Right-Amputee)	-43.16	0.0232	1.01
	24 hours	Right Antenna Only (Left-Amputee)	-31.16	0.0321	1.40
NrxI	7days	Control (Both Antennae Intact)	-14.7571	0.0678	
	7 days	Left Antenna Only (Right-Amputee)	-12.2666	0.0815	1.20
	7 days	Right Antenna Only (Left-Amputee)	-15.207	0.0658	1.03
NLG1	7 days	Control (Both Antennae Intact)	-329.317	0.0030	
	7 days	Left Antenna Only (Right-Amputee)	-257.438	0.0039	1.28
	7 days	Right Antenna Only (Left-Amputee)	-339.7508	0.0029	1.03
NLG2	7 days	Control (Both Antennae Intact)	-12.4092	0.0806	
	7 days	Left Antenna Only (Right-Amputee)	-11.4188	0.0876	1.09
	7 days	Right Antenna Only (Left-Amputee)	-12.0698	0.0829	1.06
NLG3	7 days	Control (Both Antennae Intact)	-42.4689	0.0235	
	7 days	Left Antenna Only (Right-Amputee)	-40.6918	0.0246	1.04
	7 days	Right Antenna Only (Left-Amputee)	-55.6509	0.0180	1.37
NLG4	7 days	Control (Both Antennae Intact)	-31.4139	0.0318	
	7 days	Left Antenna Only (Right-Amputee)	-25.6342	0.0390	1.23
	7 days	Right Antenna Only (Left-Amputee)	-34.0205	0.0294	1.33
NLG5	7 days	Control (Both Antennae Intact)	-60.2685	0.0166	
	7 days	Left Antenna Only (Right-Amputee)	-51.9241	0.0193	1.16
	7 days	Right Antenna Only (Left-Amputee)	-64.148	0.0156	1.06

Table S3 continued:

	GENE	AGE	CONDITION	Relative (Fold) Expression to RPL8	Relative Expression as a ratio of *RPL8*	FOLD DIFFERENCE IN EXPRESSION BETWEEN AMPUTEES AND CONTROLS	
	NrxI	14 days	Control (Both Antennae Intact)	-24.45	0.0409		
		14 days	Left Antenna Only (Right-Amputee)	-22.38	0.0447	1.09	
		14 days	Right Antenna Only (Left-Amputee)	-27.37	0.0365	1.22	
	NLG1	14 days	Control (Both Antennae Intact)	-3440.33	0.0003		
		14 days	Left Antenna Only (Right-Amputee)	-1871.53	0.0005	1.84	
		14 days	Right Antenna Only (Left-Amputee)	-479.37	0.0021	**7.18**	
	NLG2	14 days	Control (Both Antennae Intact)	-32.41	0.0309		
		14 days	Left Antenna Only (Right-Amputee)	-24.17	0.0414	1.34	
		14 days	Right Antenna Only (Left-Amputee)	-30.17	0.0331	1.07	
	NLG3	14 days	Control (Both Antennae Intact)	-72.67	0.0138		
		14 days	Left Antenna Only (Right-Amputee)	-72.25	0.0138	1.01	
		14 days	Right Antenna Only (Left-Amputee)	-64	0.0156	1.14	
	NLG4	14 days	Control (Both Antennae Intact)	-77.89	0.0128		
		14 days	Left Antenna Only (Right-Amputee)	-66.49	0.0150	1.17	
		14 days	Right Antenna Only (Left-Amputee)	-61.82	0.0162	1.26	
	NLG5	14 days	Control (Both Antennae Intact)	-94.57	0.0106		
		14 days	Left Antenna Only (Right-Amputee)	-101.48	0.0099	1.07	
		14 days	Right Antenna Only (Left-Amputee)	-101.83	0.0098	1.08	

*1 was chosen as an arbitrary value of *RPL8* expression (housekeeping gene which all experimental genes were normalised against). *Neurexin I: NrxI. Neuroligin: NLG.*
